# *Mycobacterium cajalii* sp. nov., a novel scotochromogenic rapid-growing nontuberculous mycobacterial species closely related to *Mycobacterium servetii*

**DOI:** 10.1007/s10482-026-02301-1

**Published:** 2026-04-08

**Authors:** Alexander Tristancho-Baró, Ana Milagro, Ana Isabel López-Calleja, David Badenas-Alzugaray, Natalia Burillo, Sara Sanz, Marta Guardingo, Nieves Martínez, Sofía Samper, María Jesús García, Antonio Rezusta, Jesús Viñuelas-Bayón

**Affiliations:** 1 Clinical Microbiology Laboratory, Miguel Servet University Hospital, 50009 Zaragoza, Spain; 2 Research Group On Difficult to Diagnose and Treat Infections, Institute for Health Research Aragon, Miguel Servet University Hospital, 50009 Zaragoza, Spain; 3 Biomedical and Biotechnological Sciences Program, University of La Rioja, 26006 Logroño, Spain; 4 Laboratorio de Investigación Molecular-UIT, IIS Aragón, Instituto Aragonés de Ciencias de La Salud, Hospital Universitario Miguel Servet, Pº Isabel La Católica 1-3, Planta Calle, CP 50009 Zaragoza, Aragón Spain; 5 CIBER de Enfermedades Respiratorias, Madrid, Spain; 6 Fundación IIS Aragón, Zaragoza, Spain; 7 Departamento de Medicina Preventiva y Salud Pública y Microbiología, Facultad de Medicina, Universidad Autonoma de Madrid, Madrid, Spain; 8 GEIM (Grupo de Estudio de Infecciones Por Micobacterias - SEIMC), Madrid, Spain; 9 Faculty of Medicine, Universidad de Zaragoza, 50009 Zaragoza, Spain

**Keywords:** *Mycobacterium simiae* complex, Nontuberculous mycobacteria, Phylogenomics, Pulmonary infection

## Abstract

Non-tuberculous mycobacteria (NTM) represent an expanding group of environmental microorganisms with growing clinical significance, particularly among immunocompromised patients. In this study, we describe the isolation and comprehensive characterization of a novel *Mycobacterium* species, strain HUMS_1102779-3, recovered from a sputum sample of a patient undergoing diagnostic bronchoscopy for hilar lymphadenopathy. Initial identification attempts using MALDI-TOF MS and commercial DNA∙STRIP assays were inconclusive, prompting whole-genome sequencing for comprehensive genomic and phenotypic characterization. De novo genome assembly resulted in a 6.2 Mb draft genome with a GC content of 69.03 mol% and high completeness (98.4% conserved orthologues). Phylogenetic and genomic comparisons suggest close relationship to *Mycobacterium servetii* (ANI 94.52%, dDDH 63.5%), yet below the accepted species delineation thresholds (95% and 70%, respectively), thereby supporting its status as a distinct taxon within the *Mycobacterium simiae* complex. Phylogenomic reconstruction placed HUMS_1102779-3 within a monophyletic cluster neighboring *M. servetii* and *Mycobacterium helveticum*, but genetically divergent from them. The genome encoded 5833 coding sequences, including determinants associated with antimicrobial resistance (*rbpA*, *rpoB*-like variant, *mtrA*, *efpA*), as well as virulence-related genes typical of mycobacteria, such as components of the Type VII secretion system and iron-regulated factors (*ideR*, *hbhA*). No mobile genetic elements were detected. Phenotypically, the isolate is a fast-growing, scotochromogenic, acid-fast bacillus that exhibits notable dimorphism on 7H11 agar and produce rough yellow–orange colonies after 4–5 days at 35 °C. It differs from *M. servetii* by several biochemical traits (catalase negative, Tween 80 hydrolysis positive, tellurite reduction-positive) and distinct antimicrobial susceptibility patterns, notably higher MICs for linezolid and minocycline and lower for ciprofloxacin. Based on genomic, phylogenetic, and phenotypic evidence, we propose the designation *Mycobacterium cajalii* sp. nov.,. The type strain is HUMS_1102779-3 (= CECT 31296 = DSMZ 120455), with the genome deposited under genome assembly accession number ASM4580900v1.

**Supplementary Information:**

The online version contains supplementary material available at 10.1007/s10482-026-02301-1.

## Introduction

The genus *Mycobacterium* includes two pathogens that have afflicted humankind since the dawn of history and are arguably responsible for the greatest number of infectious disease–related morbidity and mortality in history: *Mycobacterium tuberculosis* complex and *Mycobacterium leprae* (Aborghetti et al. 2025; Kim and Swaminathan 2021). The remaining species constitute a heterogeneous group collectively known as non-tuberculous mycobacteria (NTM). These organisms are widely distributed in nature, colonizing humans, animals, and diverse environmental niches such as soil and water. Some NTMs are clearly pathogenic, for example *Mycobacterium ulcerans* and *Mycobacterium kansasii*. Others, such as members of the *Mycobacterium avium* complex, *Mycobacterium abscessus*, or *Mycobacterium fortuitum*, are regarded as opportunistic pathogens. For many additional species, however, the clinical significance of their isolation from human samples remains uncertain (Sharma and Upadhyay 2020).

In recent years, the recovery of NTM has increased sharply, largely due to the introduction of liquid culture systems. Likewise, the accuracy and accessibility of identification methods have improved, initially through MALDI-TOF MS and commercial PCR assays, and more recently through advances in genomics and high-throughput sequencing technologies (Saminathan et al. 2025).

At the same time, our understanding of the pathogenic potential of these organisms has expanded substantially. Although they were once regarded as commensals or environmental contaminants with limited clinical impact, reports of disease caused by NTMs have risen, particularly among immunocompromised patients (Sharma and Upadhyay 2020). Such immunosuppression may result from natural conditions, therapeutic interventions, iatrogenic factors, or underlying infectious and neoplastic processes. The spectrum of implicated species continues to broaden as diagnostic capacity improves.

These developments pose significant diagnostic and therapeutic challenges. Correlation between in vitro susceptibility testing and clinical outcomes is frequently inconsistent, and limited clinical experience in managing these infections across varying degrees of immunosuppression further complicates treatment decisions. For these reasons, precise and reliable species-level identification is essential in all cases of NTM isolation, particularly when derived from human clinical specimens (Murthy et al. 2025).

## Materials and methods

### Culture and initial isolation

Our Microbiology Department received a sputum sample from a patient with breast cancer who was admitted to hospital for the study of an hilar lymphadenopathy using EBUS-TBNA. The presence of purulent airway secretions was noted during the procedure, accompanied by cough and respiratory symptoms in the previous days. The patient subsequently received a 6 days course of meropenem, resulting in clinical improvement, and was discharged to complete an additional 48h course of levofloxacin at home, with subsequent outpatient follow-up. Malignancy in the lymph node biopsy was excluded by further histopathological examination.

The sputum sample was processed for mycobacterial study in accordance with our laboratory routine procedures (Tristancho-Baró et al. 2025). Briefly, it was decontaminated using a solution of sodium hydroxide and N-acetylcysteine, vortexed for five minutes, and then left to stand for a further 15 min. It was then neutralised with phosphate buffer solution and concentrated by centrifugation for 20 min. The sediment was stained with auramine and seeded in BBL™ MGIT™ (Becton Dickinson) mycobacterial culture medium. It was then processed in a Becton–Dickinson Bactec™ 960 at 37 °C, following the manufacturer’s recommendations.

Once the sample tested positive, routine controls were performed on the sample: Kinyoun staining, subculturing in BD BBL™ Chocolate II agar (Becton Dickinson), and incubating at 35 °C. Subcultures were subsequently performed in Coletsos, Lowenstein-Jensen and 7H11 media and incubated at 30 °C, 35 °C and 42 °C for 5, 10 and 20 days respectively.

Our routine procedure for identifying isolated non-tuberculous mycobacteria involves performing a MALDI-TOF MS analysis (Bruker Daltonics®, Bremen, Germany) directly from the liquid medium using a protein extraction protocol that was developed in our laboratory (Fernández-Esgueva et al. 2021). If satisfactory identification is not obtained (with a minimum score of 1.85), we proceed to perform a DNA∙STRIP technology using the GenoType Mycobacterium CM and GenoType Mycobacterium AS assays (Hain Lifescience®). The identification rate obtained is 98–99% when these two techniques are used in tandem.

### Genomic analysis

#### Whole genome sequencing and quality control

A single colony of strain HUMS_1102779-3 was subcultured on two 7H11 agar plates and incubated for 4 days at 37 °C to increase the available biomass. Genomic DNA was subsequently extracted using a bead-based nucleic acid extraction on the MagCore® System (RBC Bioscience®, New Taipei City, Taiwan). The DNA yield was quantified using a Qubit fluorometer (ThermoFisher Scientific®, Walth-man, MA, USA), and library preparation was performed using the Nextera XT kit (Illumina®, San Diego, CA, USA). Sequencing was performed on a MiSeq™ platform (Illumina®, San Diego, CA, USA) using a 150 bp paired-end protocol (300 cycles). Raw read quality was assessed and filtered using the fastQC (Andrews 2010) v0.12.1 and trimmomatic (Bolger et al. 2014) v0.39 software with the following parameters: LEADING = 3, TRAILING = 5, SLIDINGWIN-DOW = 4:25, and MINLEN = 50.

For long read sequencing, high–molecular weight genomic DNA was extracted from the same subculture used for Illumina sequencing using the MTB-Complex Extraction Kit (Altair Health, Madrid, Spain), following the manufacturer’s instructions. DNA quantity and quality were assessed by spectrophotometry (NanoDrop), fluorometry (Qubit), and agarose gel electrophoresis, yielding DNA of sufficient purity, concentration, and integrity for long-read sequencing.

Oxford Nanopore Technologies (ONT) sequencing was performed using the Native Barcoding Kit and run for 72h on a MinION® (Oxford Nanopore Technologies, Oxford, UK) device. Basecalling and demultiplexing were carried out using Guppy v0.1.0, and all reads assigned to the isolate were concatenated into a single FASTQ file for downstream processing. Read filtering was performed with Filtlong v0.3.1 to improve overall data quality by removing reads shorter than 1000 bp, downsampling to a total yield of approximately 1.5 Gb, and discarding the lowest-quality 10% of reads. The quality metrics of the filtered long reads were evaluated using NanoPlot (De Coster and Rademakers 2023) v1.46.2.

#### Genome assembly and annotation

Filtered fastq files were used for de novo hybrid genome assembly following a long-reads-first assembly strategy that integrates ONT long reads with Illumina short reads to generate a high-quality consensus genome using Hybracter (Bouras et al. 2024) v0.11.2 with default parameters. The structural quality of the resulting assembly was assessed using QUAST(Gurevich et al. 2013) v5.3.0, while its functional completeness was evaluated through the identification of universal single-copy orthologues with BUSCO (Tegenfeldt et al. 2025) v6.0.0. Potential contaminant sequences in the assembly and reads were subsequently examined with GUNC (Orakov et al. 2021) v1.0.6 and Mash (Ondov et al. 2016) v2.3, respectively, using the default database for the former and the Mash sketch database (k = 21, s = 1000) built from RefSeq release 70 for the latter.

Genome annotation was performed with Prokka (Seemann 2014) v1.14.6, from which the 16S rRNA, rpoB, and hsp65 gene sequences were manually extracted. Antimicrobial resistance determinants were identified using RGI v6.0.5 in combination with CARD (Alcock et al. 2023) v 4.0.0. Abricate (Seemann 2018) v1.0.1 was used to further characterized the resistome and virulome in conjunction with MEGARES v3.0.0 and VFDB v6.0.0 databases. The strain’s pathogenic potential for humans was evaluated with PathogenFinder (Cosentino et al. 2013) v2, while the presence of mobile genetic elements was investigated using MOB-suite v3.1.9. Finally, functional annotation was carried out with Sma3s (Casimiro‐Soriguer et al. 2017) v2 and the UniRef90 database release 2025_02.

#### Genome based identification

16S rRNA gene was examined against the entire NCBI core nucleotide database using blast v2.16.0(McGinnis and Madden 2004). Alignments of individual and concatenated marker genes (16S rRNA, rpoB, hsp65) were generated with MAFFT (Katoh and Standley 2013) v7.525 against the 25 closest species identified by NCBI BLAST, in addition to the reference sequences of *M. tuberculosis* and *M. abscessus*. Phylogenies were inferred with IQ-TREE (Nguyen et al. 2015) v3.0.1 using the substitution GTR + G model, 1000 ultrafast bootstrap replicates, and 1000 approximate likelihood-ratio tests. *Corynebacterium urealyticum* was used as outgroup. Phylogenetic trees were annotated and visualized in iTOL (Letunic and Bork 2024) v7.2.2.

The taxonomic assignment was primarily based on overall genomic relatedness indices (OGRI) and phylogenomic reconstruction. The ANI of strain HUMS_1102779-3 was calculated against the type-strains of all validly published species of the genus *Mycobacterium* (until Jun-25) using ANIclustermap (Musiał et al. 2024) v2.0.1. The resulting values were organized into an ANI divergence matrix generated with an in-house script, which was subsequently used to infer a phylogeny by the Unweighted Pair Group Method with Arithmetic Mean (UPGMA) method implemented in the ape (Paradis and Schliep 2019) package v5.8.1 in R v4.4.3, using the genome of *Hoyosella altamirensis* as outgroup. Because of its high similarity, dDDH was calculated against the species of the *Mycobacterium simiae* complex using the GGDC platform. Phylogenomic analysis consisted of 56 conserved genes from different taxa and a phylogeny inferred based on the 70 most closely related *Mycobacterium* species, identified through the initial clustering analysis of genomic distances calculated with Mash, using the gcType tool (Meier-Kolthoff et al. 2022).

### Phenotypic characterization

#### Biochemical and antimicrobial properties

We performed the main biochemical reactions recommended for speciating *Mycobacterium* species, following established protocols (Brown-Elliott and Wallace 2019) including pigment production, growth at different temperatures, catalase and urease activity, nitrate and tellurite reduction, Tween 80 hydrolysis, and the ability to grow on MacConkey agar without crystal violet.

The minimum inhibitory concentrations (MICs) were determined using MIC test strips (Liofilchem®: Technical Data Sheet for MIC Test Strips for Non-Tuberculous Mycobacteria (MTS22 Rev. 0/08.02.2016) (http://www.liofilchem.net/login.area.mic/technical_sheets/MTS22.pdf). The test strips were placed on 7H11 agar with a McFarland 1 inoculum and incubated at 35 °C. The results were read after 6 and 10 days, and interpreted according to CLSI criteria (Clinical and Laboratory Standards Institute (CLSI) 2011).

#### MALDI-TOF MS

To perform the mass spectrometry profiling (MSP) of strain HUMS_1102779-3, an internal protein extraction protocol was first employed, followed by the manufacturer’s protocol (Bruker Daltonics®) for preparing custom libraries. In summary, protein extraction was performed, after which 24 spectra (three replicates in eight wells) were acquired using the mycobacterial method in FlexControl v3.4. The quality of the acquired spectra was then assessed using FlexAnalysis v3.4, and three spectra were discarded due to insufficient quality. The finalised MSP was then integrated into the custom mycobacterial MALDI-TOF library for use in future identification processes (Calderaro and Chezzi 2024).

## Results

Direct auramine staining of the sample was negative for acid-fast bacilli. Seventeen days after processing the sample, it tested positive. According to our protocol, a Kinyoun stain was performed, as well as a subculture in chocolate agar. The stain revealed acid-fast bacilli, and growth was observed on chocolate agar after 72h. Different morphotypes were observed on chocolate agar culture and were isolated by subculturing them in 7H11 media. One of these morphotypes was identified as *Mycobacterium intracellulare subsp. chimaera* with a score of 1.93 and was excluded. However, no acceptable identification could be obtained for the second isolate. Repeated attempts to identify the species using MALDI-TOF directly from liquid medium yielded inconclusive results, with *Mycobacterium interjectum*, *Mycobacterium gastri*, *Mycobacterium ilatzerense* and *Mycobacterium heckshomense* as the closest matches but neither with sufficient score for species level assignment, being 1.60, 1.55, 1.55 and 1.50 respectively. This second isolate also produced two different morphotypes in the 7H11 cultures (Fig. S1). Despite multiple attempts to obtain pure cultures of each one, the same result was always reproduced. The GenoType CM results showed bands 4 and 10, as well as band 12 in GenoType AS, in addition to the genus control band (band 3) in both techniques. These results made it impossible to identify the species, so whole genome sequencing was carried out instead. The patient responded well to empirical treatment with broad-spectrum antibiotics (linezolid and meropenem). No additional bacterial species were recovered from the sample.

ONT sequencing generated long-read data of adequate quality for hybrid assembly, with an N50 read length of approximately 7.2 kb and more than 80% of reads achieving a minimum quality score of Q20, > 95% of the short reads used for polishing were ≥ Q30. The hybrid assembly resulted in a complete and closed genome, consisting of a single circular contig of 6,205,626 bp. No plasmid sequences were detected accompanying the main chromosomal replicon. The estimated GC content was 69.03 mol%. The assembly exhibited a high level of completeness, with 98.4% conserved orthologues within the *Mycobacteriaceae* family. No contaminant sequences were detected in either the raw reads or the assembly. The genome contained 5833 coding sequences (CDS), of which 26 were catalogued as pseudogenes and 585 as hypothetical proteins. The genome also contained 3 copies of the rRNA, 51 tRNA genes, 23 ncRNAs, 1 tmRNA and 3 CRISPR array regions. The most enriched UniProt pathways were associated with lipid metabolism, amino-acid biosynthesis and cell wall biogenesis. The top three biological processes were annotated as transcription, transport and lipid metabolism and the most enriched cellular component was the membrane, all of these consistent with mycobacteria.

Four antimicrobial resistance determinants were found in HUMS_1102779-3 genome, two perfect matches and one highly similar compared to that present in *M. tuberculosis* and one highly similar in *Nocardia* spp.. Perfect matches corresponded to cell division protein regulator *mtrA* and major efflux pump *efpA*, both linked to tolerance and resistance to a broad range of antimicrobials, notably those frequently employed in the management of mycobacterial infections, including isoniazid, rifampicin, aminoglycosides, and fluoroquinolones. Sigma factor stabilization RNA Polymerase-binding Protein A (*rbpA*) conferring resistance to rifamycins, was found with a similarity of 96.2%. Finally, a variant of rpoB present in *Nocardia* spp. conferring resistance to rifamycins was found with an identity of 82%. Several virulence factors related to Mycobacterium tuberculosis were found. The most prevalent were genes related to Type VII secretion system, although other loci related to iron metabolism such as iron-dependent repressor *IdeR* and iron-regulated heparin binding hemagglutinin *hbhA* were also found alongside various secreted antigens and heat shock proteins. No mobile genetic elements were found. PathogenFinder2 found HUMS_1102779-3 as probable human pathogen with a high confidence average score of 0.9 (with ¾ of neural networks yielding a score greater than 0.93).

**Fig. 1:**
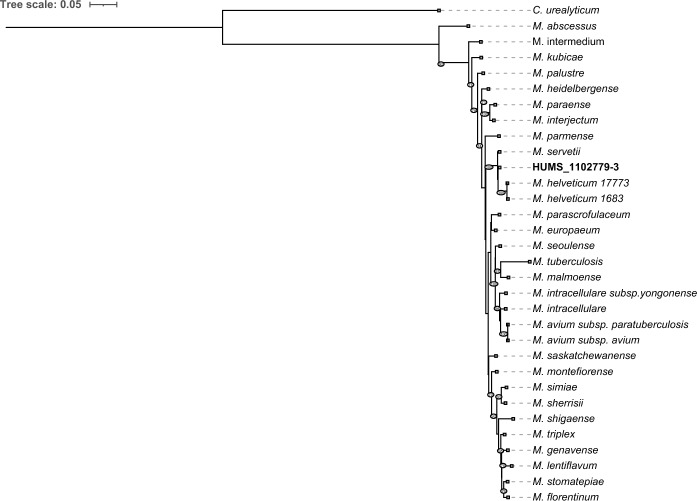
Inferred phylogeny of closely related Mycobacterium species using the concatenated alignment of 16S rRNA, rpoB and hsp65 genes

**Fig. 2:**
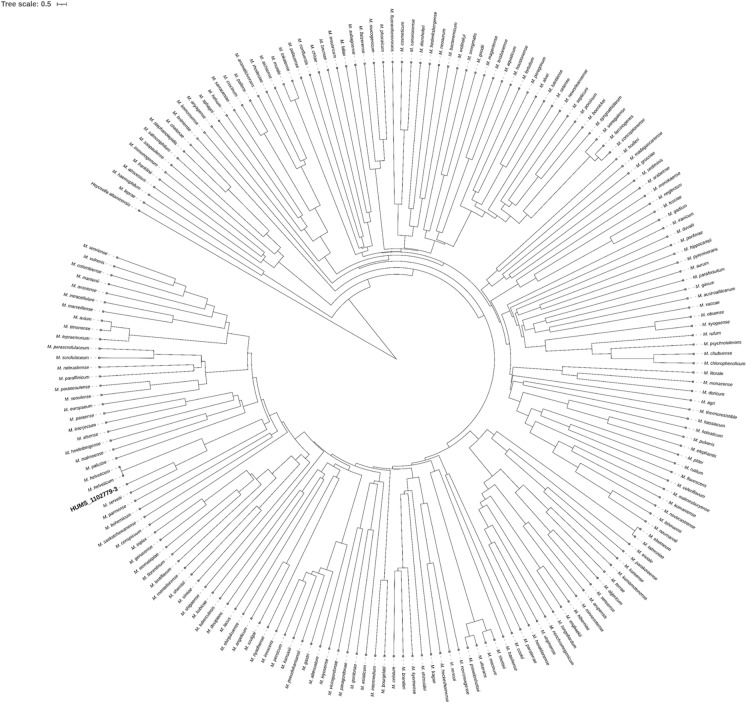
ANI based phylogeny using UPGMA algorithm

**Fig. 3:**
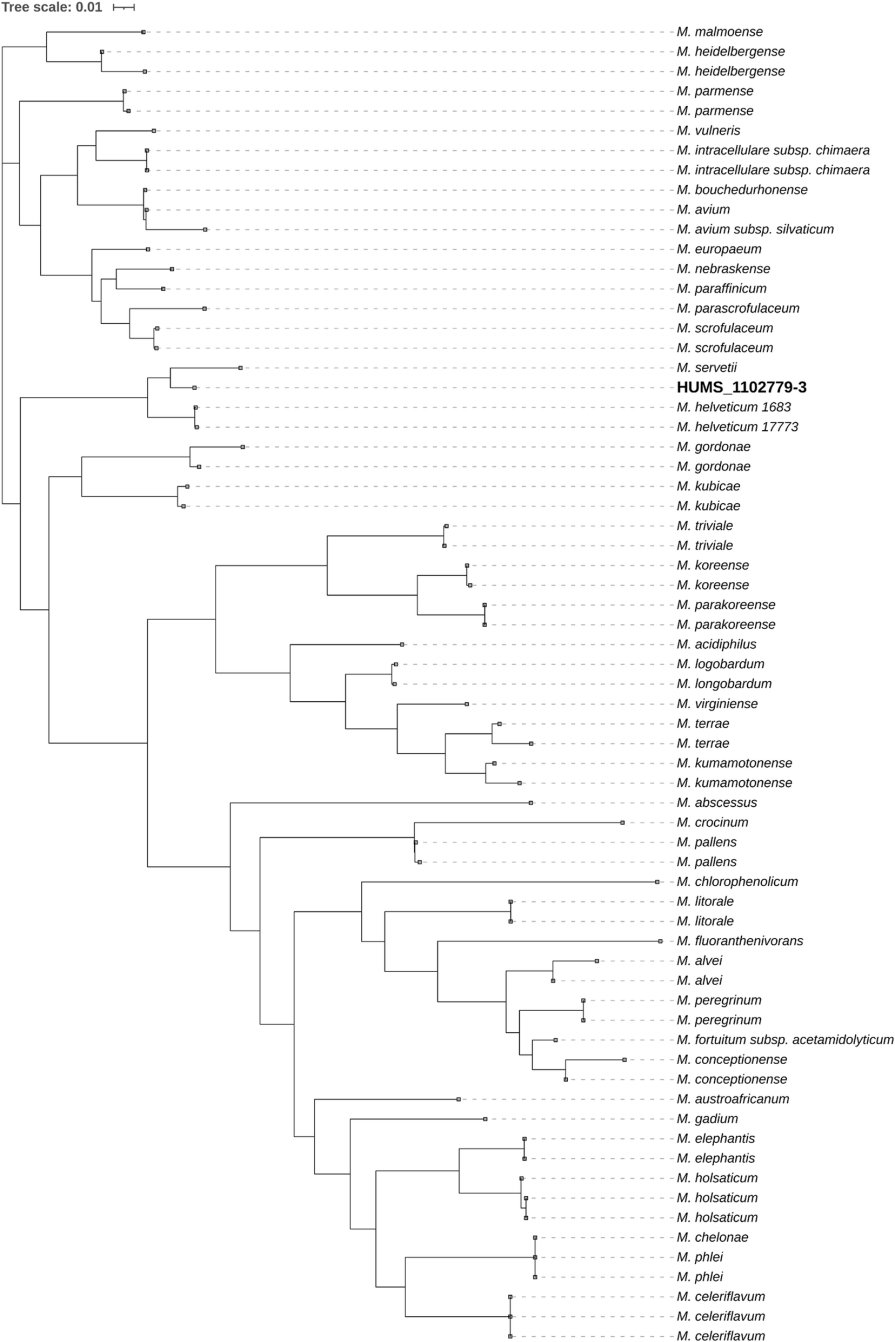
Phylogenomic reconstruction of closely related taxa using 56 conserved loci

16S ribosomal gene blast analysis presented *Mycobacterium servetii*, *Mycobacterium helveticum* and *Mycobacterium florentinum* as closely related species with 99.8%, 99.6% and 98.9% identity, respectively. Marker genes phylogenies (including the concatenated three marker genes) consistently placed HUMS_1102779-3 as an independent clade, but very closely related to *M. servetii* and *M. helveticum*, with the exception of *hsp65*, in which it is placed at same level of *M. servetii*, probably due to short sequence length and lower genetic discriminatory power. Marker genes phylogenies also accurately clustered HUMS_1102779-3 within the *M. simiae* complex (Fig. 1). Despite its high identity values using traditional marker genes analysis, OGRI analysis presented HUMS_1102779-3 as an independent new taxon. Besides, complete genome sequence comparison clearly identifies HUMS_1102779-3 as a separate species. First, ANI values against all validly published *Mycobacterium* species as of June 2025, remained below the 95% threshold. Highest values were found for *M. servetii*, *M. helveticum* and *M. heidelbergense*, rising up to 94.3%, 90.6% and 84.0%, respectively. UPGMA phylogeny based on ANI results is shown in Fig. 2. Second, all dDDH values of HUMS_1102779-3 against the top 10 ANI-related species were below the 70% threshold, with *M. servetii*, *M. helveticum* and *Mycobacterium europaeum* being the closest matches with 63.5%, 54.8% and 36.8%, respectively (Table 1). Finally, phylogenomics analysis presented HUMS_1102779-3 within the same cluster but genetically divergent from *M. servetii* and forming a monophyletic clade including *M. helveticum* as shown in Fig. 3. Values below 95% and 70% (ANI and dDDH respectively) identify different bacterial species by genome comparison(Meier-Kolthoff et al. 2014; Riesco and Trujillo 2024).

**Table 1:** OGRI analysis summary of closely related Mycobacterium species against HUMS_1102779-3

Taxa	ANI	dDDH
*M. servetii* 12766410 HUMS	94.52	63.50
*M. helveticum* s17773	90.69	54.80
*M. heidelbergense* JCM14842	84.20	33.50
*M. malmoense* ATCC29571	83.97	34.00
*M. europaeum* CSRUP1344	83.84	36.80
*M. saskatchewanense* JCM13016	82.91	31.10
*M. florentinum* JCM14740	82.30	27.20
*M. stomatepiae* JCM17783	81.97	26.70
*M. montefiorense* BS	81.63	25.90
*M. triplex* DSM44626	81.27	27.60

Once the genomic individual traits of the new taxon were established, a comprehensive phenotypic characterization was performed. The findings of an antimicrobial susceptibility study against antibiotics commonly used in the treatment of infections caused by rapidly growing mycobacteria is shown in Table 2. Table 3 summarizes the main biochemical properties compared to closely related mycobacterial species.

**Table 2:** Antimicrobial susceptibility profile of HUMS_1102779-3

	Concentration range tested (mg/L)	6 days (mg/L)	10 days (mg/L)	Interpretation
Amikacin	0.016–256	2	4	S
Ciprofloxacin	0.002–32	0.032	0.125	S
Clarithromycin	0.016–256	0.064	0.064	S
Cotrimoxazole	0.002–32	> 32	> 32	R
Linezolid	0.016–256	32	> 256	R
Minocycline	0.016–256	16	> 256	NI
Moxifloxacin	0.002–32	0.016	0.032	S
Rifampicin	0.016–256	0.125	0.25	NI

**Table 3:** Biochemical properties of HUMS_1102779-3

Biochemical features	1	2	3
Pigmentation (scotochromogenic)	+	+	+
Growth at 30 °C	+	+	+
Growth at 35 °C	+ +	+	+
Growth at 42 °C	± (poor growth)	+	−
Growth in less than 7 days	+	+	−
Growth in MacConkey agar without crystal violet	−	−	−
Thermostable catalase	−	+	+
Semi-quantitative catalase	−	+	+
Nitrate reduction	−	−	−
Urease	−	−	−
Tween 80 hydrolysis	+	−	−
Tellurite reduction	+	−	+

1: HUMS_1102779-3; 2: M. *servetii*; 3: M. *helveticum*.

Although results are derived from a single isolate, the antibiotic susceptibility is presented in tabular format to facilitate clarity and readability, given that multiple antimicrobial agents were tested and the data are directly compared with those of the closest related species so far.

**Fig. 4:**
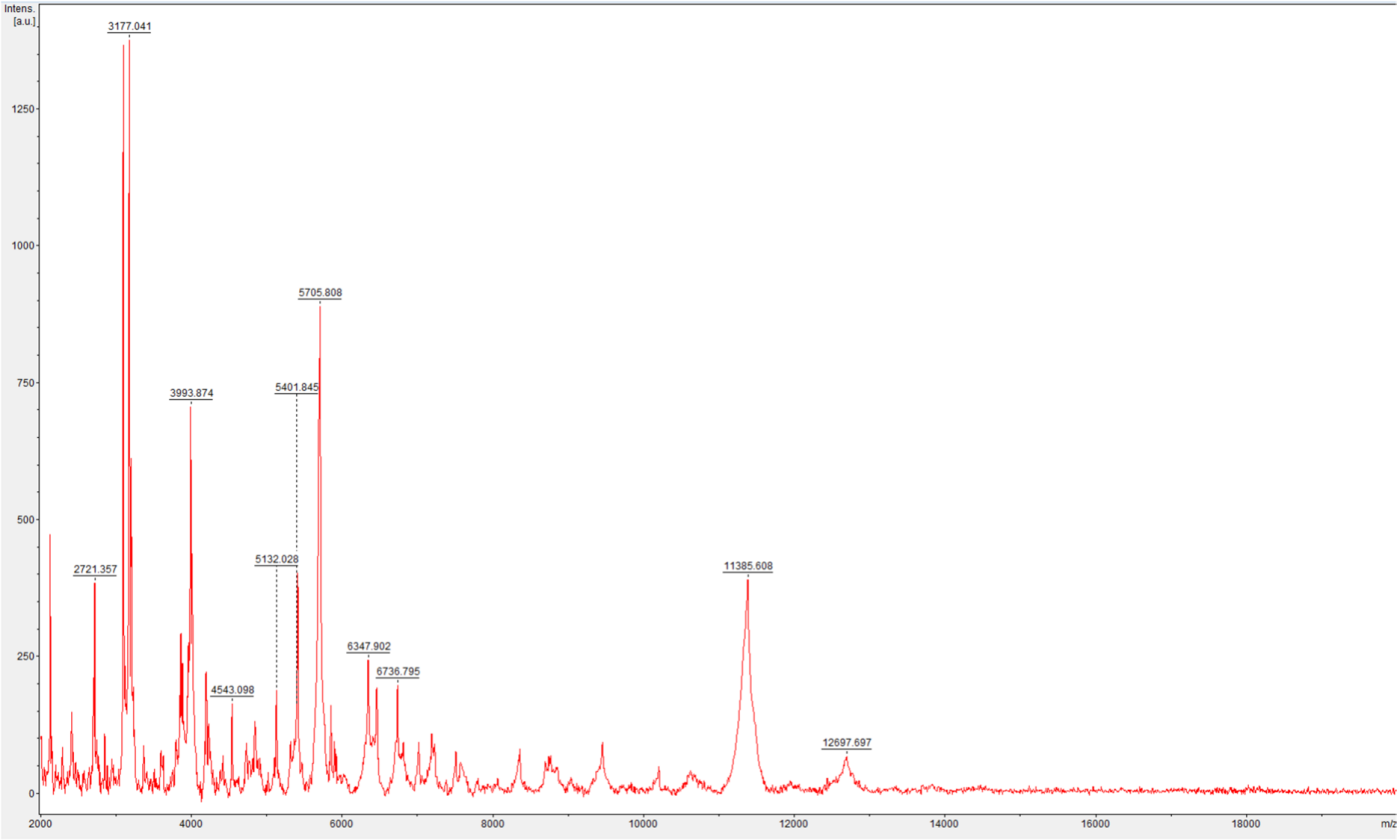
MSP of HUMS_1102779-3

The MSF spectrum of MALDI-TOF MS was subsequently characterized (Fig. 4), and its accuracy and specificity were further evaluated using concurrent evaluation involving other *Mycobacterium* species in the same MALDI-TOF run, including *M. servetii*. Importantly, no cross-identifications were observed (Fig. S2).

## Discussion

Strain HUMS_1102779-3 was isolated from a human sputum sample after seventeen days of incubation in BBL™ MGIT™. It exhibits morphological and growth characteristics consistent with those of mycobacteria; however, all routine microbiological analyses proved inconclusive for species-level identification. Genomic identification analyses, performed against the type-strains genomes of all validly published *Mycobacterium* species (Until Dec-24), strongly supports the designation of HUMS_1102779-3 as a novel taxon, based on the following criteria: (i) its independent phylogenetic clustering in gene-based analyses; (ii) an ANI value below 95%; (iii) a dDDH value below 70%; and (iv) a clear phylogenomic divergence (Riesco and Trujillo 2024). Notably, the OGRI values and genetic distances inferred from less discriminative phylogenies fall close to the lower boundary of the species delineation threshold or cluster together with other taxa, particularly *M. servetii*, reflecting its evolutionary proximity. *M. servetii* is a recently identified *Mycobacterium* species, described at the same laboratory (Tristancho-Baró et al. 2025); nevertheless, several phenotypic traits provide robust evidence for the recognition of HUMS_1102779-3 as an independent taxon as well. These include: (i) its shorter growth time at 35 ºC compared with *M. servetii*; (ii) distinctive colony morphology on solid agar media (HUMS_1102779-3 colonies are rough and dimorphic, whereas *M. servetii* produces smooth, creamy, and well-defined colonies); (iii) unique hybridization banding patterns; (iv) divergent biochemical properties (HUMS_1102779-3 is catalase negative, tween 80 hydrolysis positive and reduces tellurite in opposition to *M. servetii*); (v) distinct antimicrobial susceptibility profiles (High MICs against linezolid and minocycline and reduce MIC against Ciprofloxacin in comparison to *M. servetii*); and (vi) a reproducible MSP, which enables reliable discrimination between the two species using MALDI-TOF MS.

While the proportion of smear-positive cases among patients with tuberculosis ranges between 50 and 60%, this percentage drops sharply to around 10% in cases of non-tuberculous mycobacteria (NTM). Therefore, the negative result of the direct auramine stain in this sample is fully consistent with these epidemiological data and does not, in any way, invalidate the subsequent isolation of strain HUMS_1102779-3 in culture. Indeed, most NTM isolates from clinical specimens are recovered precisely in the context of negative direct microscopy results (auramine or Ziehl–Neelsen) (Donkeng Donfack et al. 2024).

It is noteworthy that phylogenetic analyses consistently place HUMS_1102779-3 and its closest relative, *M. servetii*, within clades predominantly composed of slow-growing NTM, despite both taxa exhibiting a rapid growth phenotype. Although uncommon, this phenomenon has been previously described. Notably, Tortoli et al. demonstrated that the *Mycobacterium chelonae–abscessus* complex, a well-recognized group of rapidly growing NTM, occupies one of the most ancestral phylogenetic positions and may exhibit closer evolutionary relationships to certain slow-growing species than to other rapidly growing taxa (Tortoli et al. 2017). This apparent discrepancy can be partially explained by the fact that phylogenetic reconstructions based on ANI or conserved core genes primarily reflect genome-wide similarity rather than specific phenotypic traits such as growth rate. Growth dynamics in NTM are driven by the presence or absence of particular genetic determinants, including operons and gene families involved in nutrient uptake and cellular physiology, such as the livFGMH operon, ABC transporters, the shaACDEFG gene cluster, and the MspA porin (Bachmann et al. 2020).

Accordingly, a species may retain high overall genomic similarity to slow-growing mycobacteria while possessing (or reacquiring) the specific genetic repertoire required for rapid growth. This may explain why phylogenetic positioning does not always align with the observed growth phenotype. Such gene acquisition events could be mediated by evolutionary processes, including horizontal gene transfer HGT or larger genome rearrangements, which may introduce or restore growth-related genetic elements within a given lineage, thereby decoupling phenotype from core genome phylogeny (Zhang et al. 2023).

Conversely, the transition from rapid to slow growth has been associated primarily with gene family contraction, particularly affecting ABC transporters involved in amino acid and ion transport, as well as quorum sensing related genes, rather than large-scale genome restructuring. The same principle applies for genetic expansion and phenotypic trait restore (Zhu et al. 2023). Intermediate evolutionary states may therefore exist, in which partial gene loss results in phenotypic shifts without substantial alteration of the overall genomic backbone. In such cases, a species may cluster phylogenetically with either rapid- or slow-growing groups despite exhibiting a divergent growth phenotype.

Finally, it is important to consider that ANI-based metrics and core-genome phylogenies are optimized for taxonomic resolution and may not reliably predict phenotypic traits. Two species may display high ANI values while differing substantially in the presence or absence of specific operons governing growth rate. Consequently, a rapidly growing species whose core genome is phylogenetically nested within slow-growing lineages may still exhibit high ANI to those taxa, irrespective of its growth characteristics (Zhang et al. 2023).

Interestingly, strain HUMS_1102779-3 exhibits a distinctive pleomorphism in its colony morphology when grown on 7H11 medium. Although variations in morphology have been previously reported in several *Mycobacterium* species depending on culture age (Ramesh et al. 2025), and distinct morphotypic variants have been described within the same NTM species (Fregnan and Smith 1962; Pichler et al. 2024), the dimorphic pattern observed within a single culture of HUMS_1102779-3 is particularly remarkable. This phenomenon may lead to difficulties in its recognition in clinical samples, as mixed or atypical morphologies could easily be misinterpreted as contamination or polymicrobial growth. These findings highlight the relevance of considering phenotypic variability during the diagnostic and taxonomic identification of NTM, especially in cases where unusual or unstable morphologies are observed.

Although only a single isolate is currently available, the fact that HUMS_1102779-3 was recovered in the absence of other respiratory pathogens, from a patient presenting compatible symptoms and predisposing factors for NTM infection (Chai et al. 2022), strongly suggests that this strain is of clinical significance. However, additional isolates and further studies are required to confirm its pathogenic role, as well as to clarify its possible role within the respiratory microbiota. Taking all these factors into account, its thorough characterization and formal recognition as a novel NTM species are justified, as this will facilitate its identification and recovery by other clinical laboratories and promote further research into its pathogenic potential and ecological niche.

Together, these findings underscore the importance of describing HUMS_1102779-3 as a distinct species within the genus *Mycobacterium*, contributing to a more accurate understanding of the diversity and clinical impact of NTM.

## Description of *Mycobacterium cajalii* sp. nov.

In honor of the great Spanish physician, bacteriologist, histologist and pathologist, Santiago Ramón y Cajal—considered the father of neuroscience and recipient of the 1906 Nobel Prize in Medicine for his contributions to the understanding of the structure of the nervous system—we propose the name *Mycobacterium cajalii* (ca.ja’li.i. N.L. gen. n. cajalii [italics], of Cajal). Isolate HUMS_1102779-3 is a scotochromogenic, fast-growing, non-motile, non-spore-forming species of Mycobacterium. Microscopically is a rod-shaped, acid-fast bacillus with variable size (Fig. S3) depending on culture age (incubation length). Growth requires four to five days of incubation, during which time a yellow pigment develops that becomes more intense over a few days and takes on an orange hue with lengthier incubations (Fig. S4). Marked dimorphism is observed in 7H11 cultures. Antimicrobial susceptibility testing revealed low MICs for amikacin, clarithromycin, moxifloxacin, ciprofloxacin and rifampicin and high MICs to cotrimoxazole, minocycline and linezolid. The annotated draft genome of strain HUMS_1102779-3 comprises 6,205,626 bp, which has been deposited in a public database. This Whole Genome Shotgun project has been submitted to the DDBJ/ENA/GenBank database under the accession number PRJNA1192957. The genome assembly accession described in this article is version ASM4580900v1. The GenBank accession number for the 16S rRNA gene is PV889360.1. The type strain is HUMS_1102779-3 (= CECT 31296, = DSMZ 120455).

## Supplementary Information

Supplementary file1. Fig. S1 Colony dimorphism on 7H11 media agar (DOCX 22 KB)

Supplementary file2. Fig. S2 MALDI-TOF MS identification results including HUMS_1102779-3 among other isolates (DOCX 22 KB)

Supplementary file3. Fig. 3 Cell size variation according to culture age (DOCX 22 KB)

Supplementary file4. Fig. 4 Colony pigment after prolonged incubation (DOCX 22 KB)

## Data Availability

This Whole Genome Shotgun project has been submitted to the DDBJ/ENA/GenBank database under the accession number PRJNA1192957. HUMS_1102779-3 has been deposited into the CECT and DSMZ collections under accession numbers 31296 and 120455 respectively.

## Abbreviations

PCR: Polymerase chain reaction
MALDI-TOF MS: Matrix-assisted laser desorption/ionization time-of-flight mass spectrometry
EBUS-TBNA: Endobronchial ultrasound-guided transbronchial needle aspiration
ANI: Average nucleotide identity
dDDH: Digital DNA–DNA hybridization
MAC: *Mycobacterium avium-intracellulare* Complex
MSP: Mass spectra profile
NTM: Nontuberculous mycobacteria
OGRI: Overall genome relatedness index
UPGMA: Unweighted pair group method with arithmetic mean
CLSI: Clinical Laboratory Standards Institute
