# Supplementary figures and images for: Mycobacterium cajalii sp. nov., a novel scotochromogenic rapid-growing nontuberculous mycobacterial species closely related to Mycobacterium servetii
Source: Antonie Van Leeuwenhoek. 2026 Apr 8;119(5):92. doi: 10.1007/s10482-026-02301-1 (PMC13061764; doi:10.1007/s10482-026-02301-1)

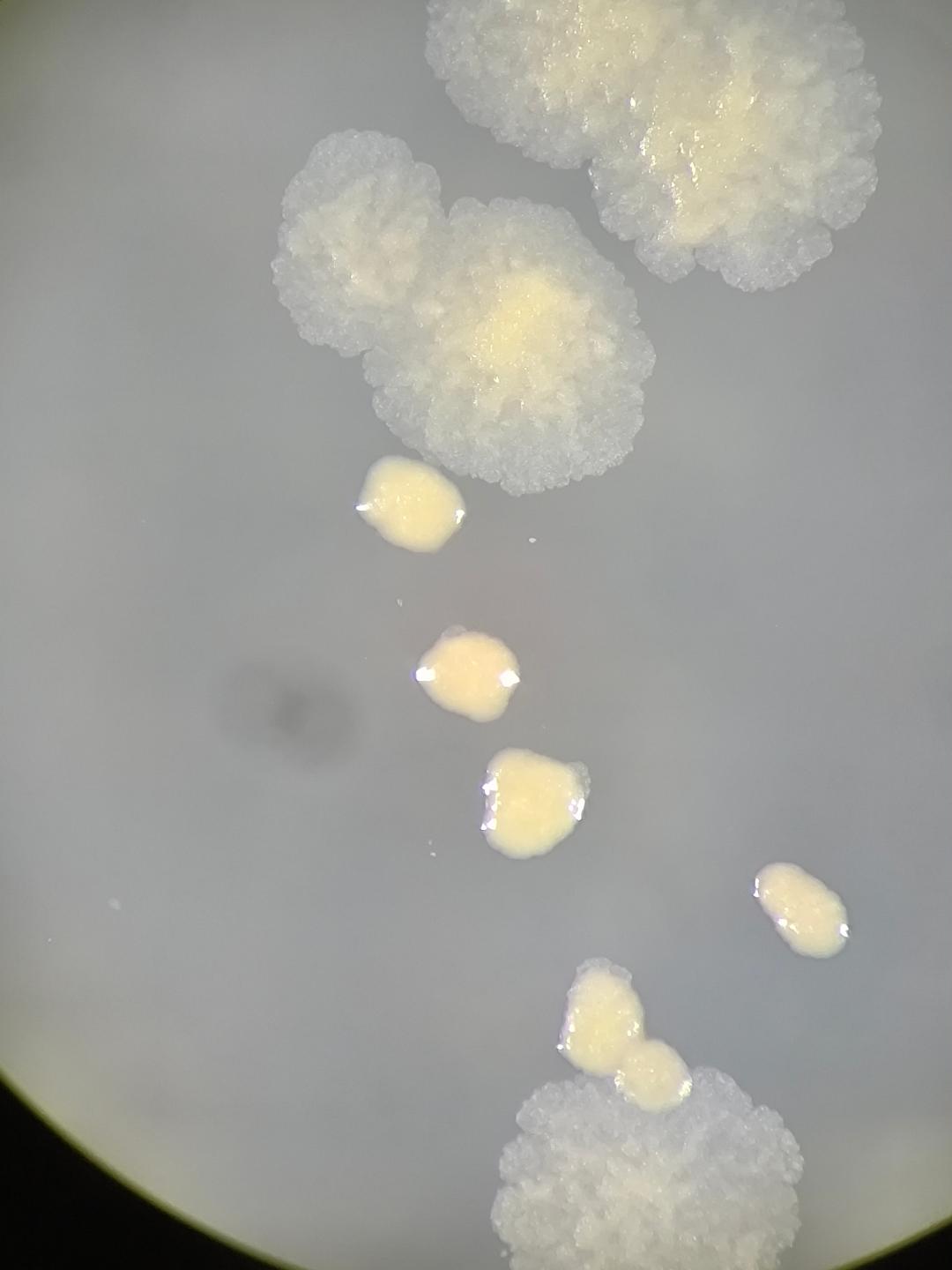

Supplement: Supplementary file 1 — Supplementary file1. Fig. S1 Colony dimorphism on 7H11 media agar (DOCX 22 KB) [file 10482_2026_2301_MOESM1_ESM.jfif]

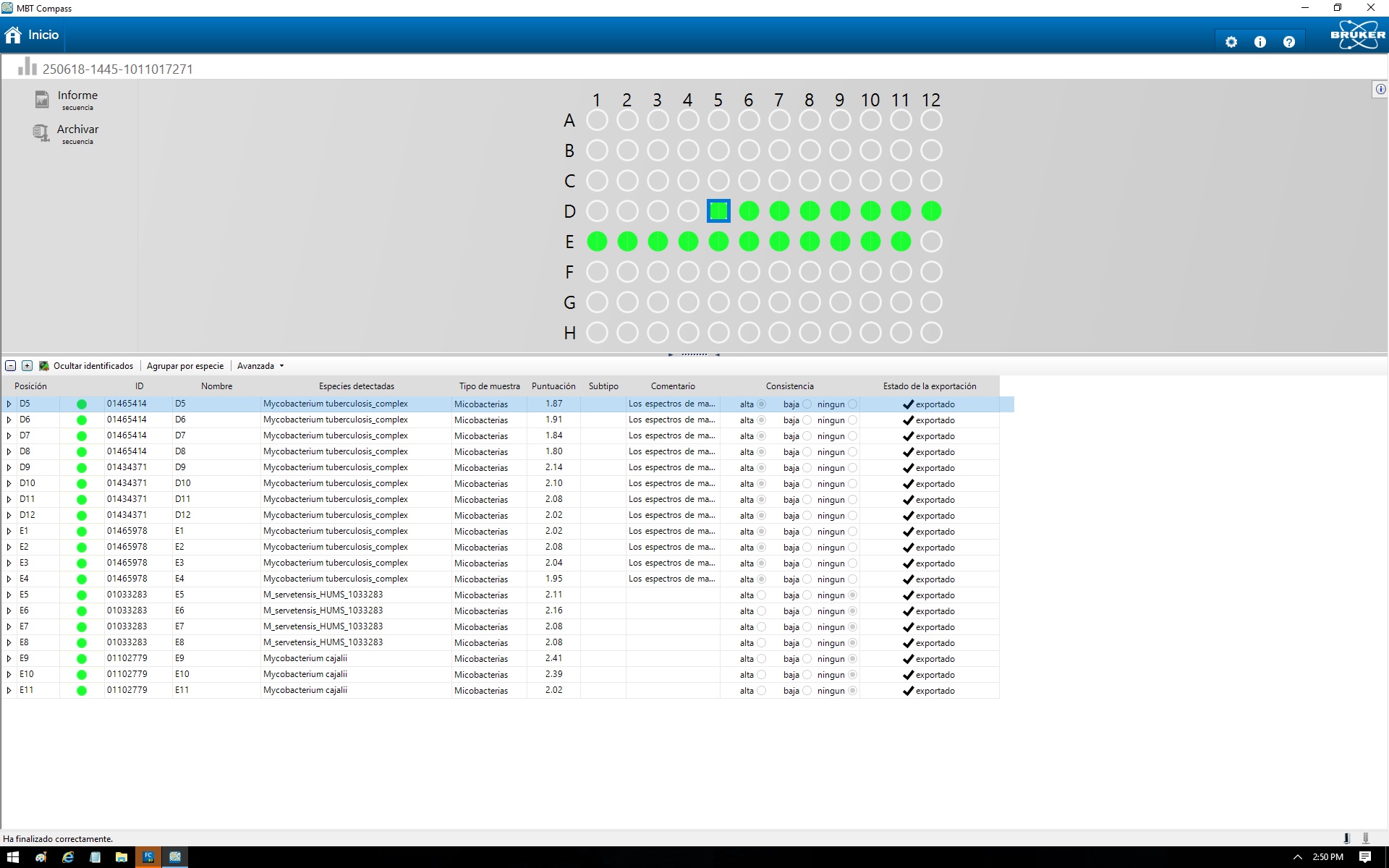

Supplement: Supplementary file 2 — Supplementary file2. Fig. S2 MALDI-TOF MS identification results including HUMS_1102779-3 among other isolates (DOCX 22 KB) [file 10482_2026_2301_MOESM2_ESM.jpg]

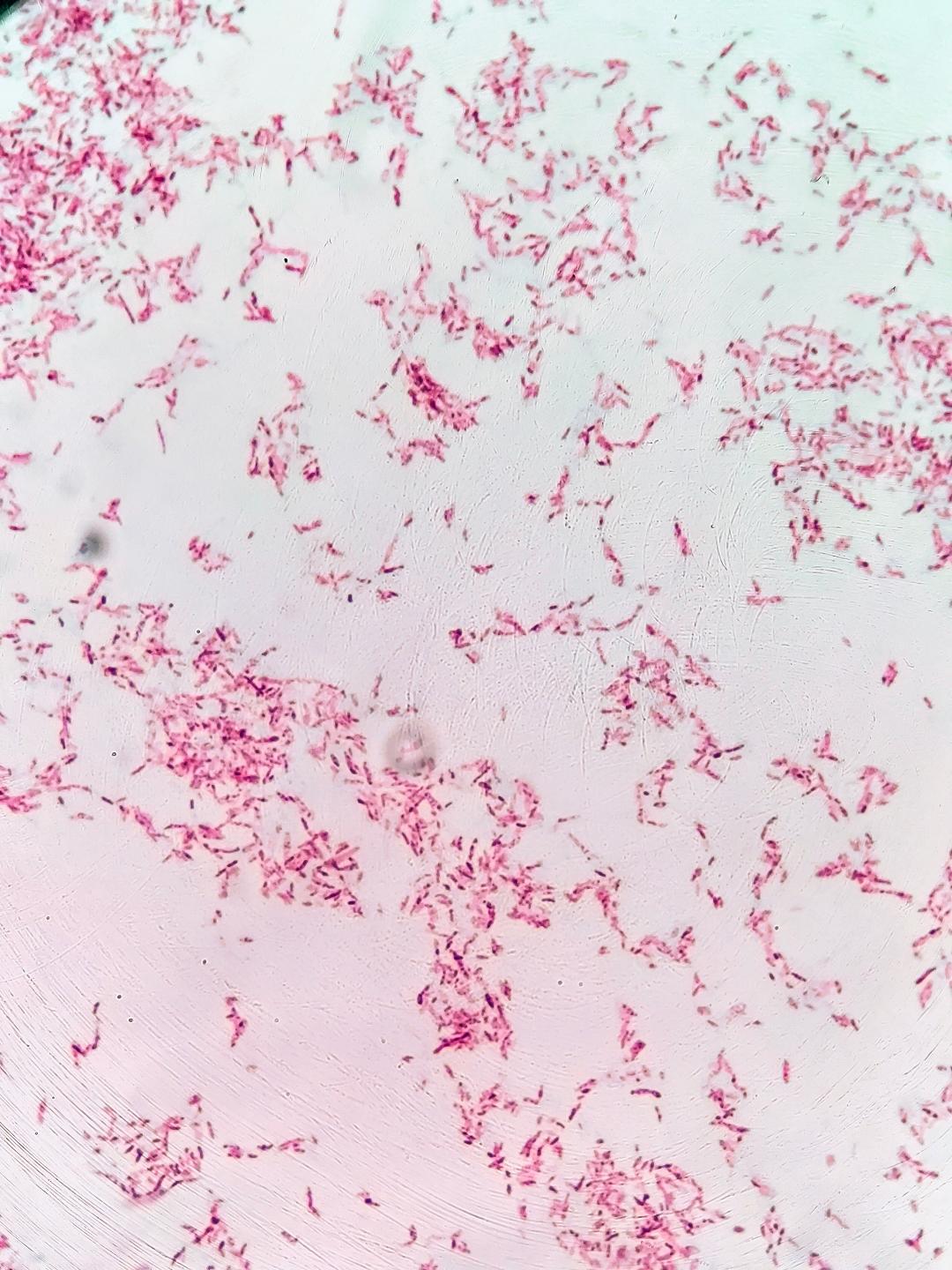

Supplement: Supplementary file 3 — Supplementary file3. Fig. 3 Cell size variation according to culture age (DOCX 22 KB) [file 10482_2026_2301_MOESM3_ESM.jfif]

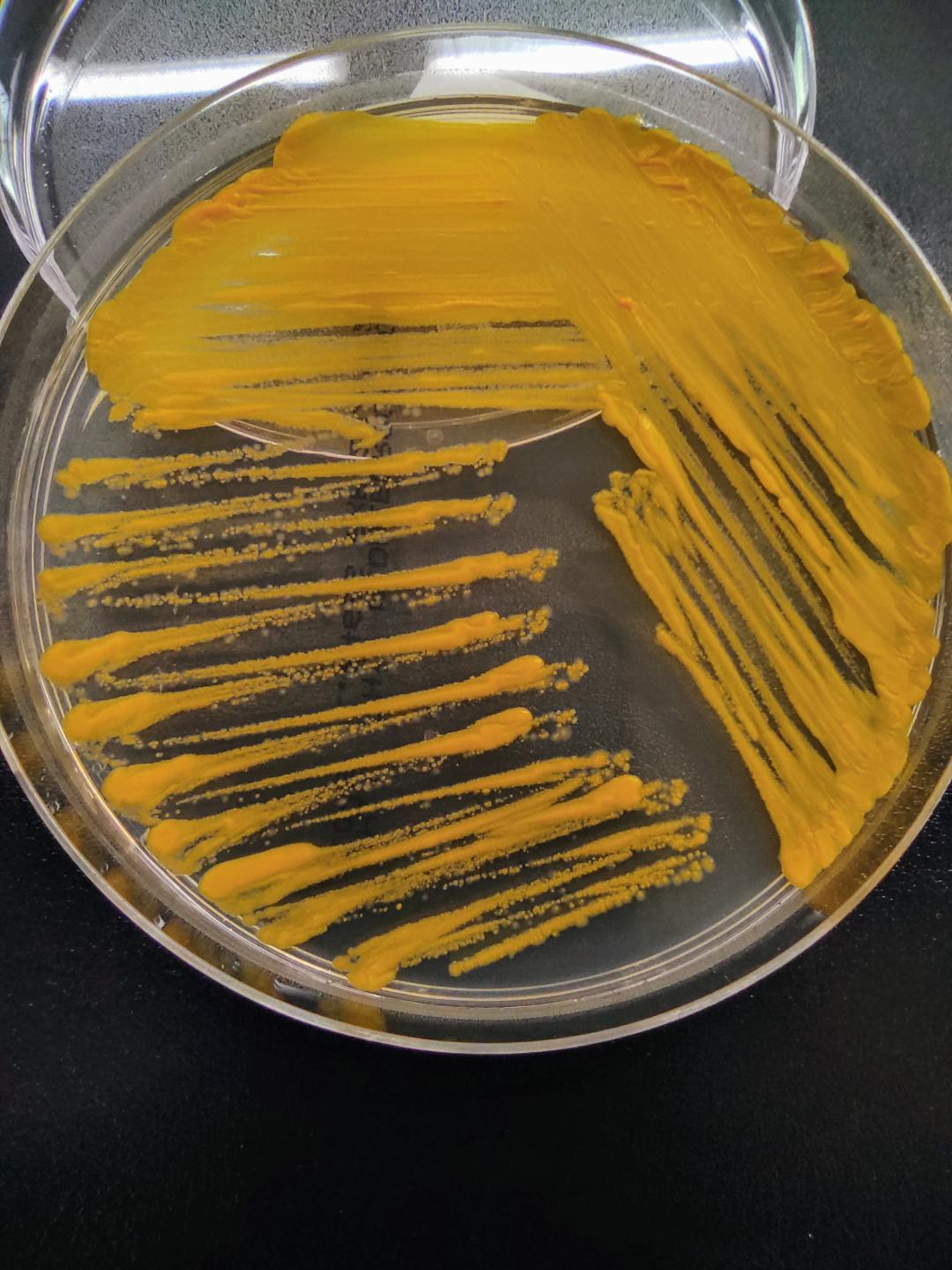

Supplement: Supplementary file 4 — Supplementary file4. Fig. 4 Colony pigment after prolonged incubation (DOCX 22 KB) [file 10482_2026_2301_MOESM4_ESM.jfif]
